# Predicting mortality after start of long-term dialysis–International validation of one- and two-year prediction models

**DOI:** 10.1371/journal.pone.0280831

**Published:** 2023-02-22

**Authors:** Mikko Haapio, Merel van Diepen, Retha Steenkamp, Jaakko Helve, Friedo W. Dekker, Fergus Caskey, Patrik Finne

**Affiliations:** 1 Department of Nephrology, University of Helsinki and Helsinki University Hospital, Helsinki, Finland; 2 Department of Clinical Epidemiology, Leiden University Medical Center, Leiden, The Netherlands; 3 UK Renal Registry, Bristol, United Kingdom; 4 Finnish Registry for Kidney Diseases, Helsinki, Finland; 5 Population Health Sciences, University of Bristol, Bristol, United Kingdom; Istituto Di Ricerche Farmacologiche Mario Negri, ITALY

## Abstract

**Background:**

Mortality prediction is critical on long-term kidney replacement therapy (KRT), both for individual treatment decisions and resource planning. Many mortality prediction models already exist, but as a major shortcoming most of them have only been validated internally. This leaves reliability and usefulness of these models in other KRT populations, especially foreign, unknown. Previously two models were constructed for one- and two-year mortality prediction of Finnish patients starting long-term dialysis. These models are here internationally validated in KRT populations of the Dutch NECOSAD Study and the UK Renal Registry (UKRR).

**Methods:**

We validated the models externally on 2051 NECOSAD patients and on two UKRR patient cohorts (5328 and 45493 patients). We performed multiple imputation for missing data, used c-statistic (AUC) to assess discrimination, and evaluated calibration by plotting average estimated probability of death against observed risk of death.

**Results:**

Both prediction models performed well in the NECOSAD population (AUC 0.79 for the one-year model and 0.78 for the two-year model). In the UKRR populations, performance was slightly weaker (AUCs: 0.73 and 0.74). These are to be compared to the earlier external validation in a Finnish cohort (AUCs: 0.77 and 0.74). In all tested populations, our models performed better for PD than HD patients. Level of death risk (i.e., calibration) was well estimated by the one-year model in all cohorts but was somewhat overestimated by the two-year model.

**Conclusions:**

Our prediction models showed good performance not only in the Finnish but in foreign KRT populations as well. Compared to the other existing models, the current models have equal or better performance and fewer variables, thus increasing models’ usability. The models are easily accessible on the web. These results encourage implementing the models into clinical decision-making widely among European KRT populations.

## Introduction

As end-stage kidney disease (ESKD) becomes more common, there is an expanding need for long-term kidney replacement therapy (KRT) [1]. To plan future nephrological resources, it is important to be able to predict outcome of KRT patients. In addition, to make sound and justified treatment decisions, which are shared with individual ESKD patients, we need personalized outcome prediction.

In KRT patients, survival may be considered as the primary outcome of the treatment. To predict survival, or risk of death, several mathematical models have been constructed [2–12] but none have gained popularity in clinical work. This may be because they have not been found practical to use or benefit from their use is unclear. More importantly, for most of the models, generalizability and hence reliability in new KRT populations is unknown.

Validation of a prediction model externally and internationally, in an KRT patient population of another country than the one used for model construction, is key to finding the best performing models suitable for international clinical application [13–15]. This work has recently begun and is already revealing some differences in models’ performance [12,16–19]. So far, however, most published models only perform moderately, and a model performing clearly better than the others has not been found.

We have previously published two mortality prediction models based on Finnish patients starting long-term dialysis [20]. These models are promising, include only six to seven variables, and performed well when externally (temporally) tested on a Finnish KRT population [20]. The aim of this study is to evaluate whether these models possess prediction capability within a broader, more recent, and international context. The Dutch NECOSAD (the Netherlands Cooperative Study on the Adequacy of Dialysis) study [21] and the United Kingdom Renal Registry (UKRR) [22] were chosen as suitable validation cohorts, as these databases are of high quality and comprehensive in terms of clinical data. Should the models’ performance be good in this international validation, their adoption into clinical management of ESKD patients not only in Finland but more widely in Europe could be considered, to inform nephrologists and their patients in shared treatment decision-making for patients starting long-term KRT.

## Materials and methods

### Data source and study populations

One- and two-year all-cause mortality prediction algorithms were constructed using logistic regression based on data from the Finnish Registry for Kidney Diseases [23], and this has been described elsewhere [20]. The variables included in both prediction models were age, primary kidney disease diagnosis, heart failure, level of serum albumin and of serum C-reactive protein. The one-year model further included peripheral vascular disease and serum phosphate, while the two-year model included peripheral vascular disease with limb amputation. We validated the two prediction models temporally in a later Finnish cohort of patients who entered dialysis in 2009 to 2012 (*n =* 1768) [20]. For comparison, these earlier published validation results are also shown in the current study.

We now performed validation of the above-mentioned prediction algorithms in data of patients from the Dutch NECOSAD study and the UK Renal Registry. The NECOSAD population has been extensively investigated in numerous studies [24]. NECOSAD is a multicenter, observational, prospective cohort study which included consecutive incident dialysis patients (*n* = 2051) between January 1997 and April 2007, in the 38 participating (out of a total 49) dialysis centers in the Netherlands, with follow-up data on death available until 1 February 2015 [25]. In the present study, length of follow-up was one or two years from KRT start, depending on which prediction model we tested. From the total of 2051 patients, we excluded 87 patients from the one-year analysis and additional 51 from the two-year analysis, thus leaving 1964 and 1913 patients in the one- and two-year validation cohorts, respectively. The reasons for exclusion were (*n* for one-year model / *n* for two-year model): 1) kidney recovery before 90 days on dialysis (8/8), 2) patient refusal (62/87), 3) loss to follow-up (3/7), 4) research center ceased to take part in the study (0/14), and 5) other (14/22, both numbers including those six for whom data on age and gender were not available).

The UKRR is a non-profit organization and part of the Renal Association in the UK [26]. The UKRR is acknowledged for having high quality clinical databases, for instance, with regard to ESKD patients’ comorbidities [27]. For the validation, we included all patients who started dialysis between 1 January 2009 and 31 December 2015, a total of 45493 patients. Altogether, 71 renal centers treating adult patients in the UK reported their data to UKRR. We identified 11 centers with exceptionally high availability of data on model variables, and we named this cohort UKRR-1. We also tested the models’ performance on the entire UKRR cohort, named UKRR-2 cohort. Follow-up of patients was one or two years, again depending on the model being tested, as for the NECOSAD cohort.

In all the validation cohorts we included those dialysis patients whose hemodialysis or peritoneal dialysis had lasted at least 3 months. The patients for whom dialysis was started but who died within 3 months from the start were included. The patients who regained their kidney function in less than 3 months from dialysis start were excluded, as their treatment was not considered long-term. The validation groups also included patients who received kidney transplant after commencing dialysis treatments, and in none of the cohorts patients were censored at time of kidney transplantation.

### Data ethics statement

Our study is based on information retrieved from national kidney registries and the Dutch NECOSAD Study database. No intervention was performed, and the patients were not contacted. In the countries included in the study, registry-based studies where patients are not contacted do not require approval by an ethical review board. Nonetheless, for the NECOSAD Study, the Medical Ethical Committee of the Amsterdam University Medical Center approved the protocol. The processing of UKRR data for research has been approved by the NRES Committee North East—Newcastle & North Tyneside 1 Research Ethics Committee, reference 21/NE/0045.

All the patients included in the Finnish Registry for Kidney Diseases and the NECOSAD Study database had given written informed consent that their information may be used for research purposes. For UKRR data, a waiver of consent for research purposes has been granted centrally by the Health Research Authority, reference 16/CAG/0064.

No minors were included in this study.

### Data collection time-point

The Finnish laboratory data were collected just before dialysis commencement. In the NECOSAD study, serum albumin was measured at start of dialysis, and the rest at three months from dialysis start. In the UKRR, serum albumin and phosphorus and blood hemoglobin were measured quarterly, so these data originated from the period from dialysis start to maximum of three months after start. For other than laboratory data, the collection time-point was dialysis start in all data sets used in this study.

### Data completeness

The Finnish validation set was at least 91% complete for all variables in the prediction model (Table 1). In the NECOSAD cohort, data were 88–100% complete for all variables except for C-reactive protein (41%). In the two UKRR cohorts, data were complete for age and outcome, but for C-reactive protein data were available only for selected patients (with indication to measure it), and thus we did not use those data. Instead, for all patients in the two UKRR cohorts we decided to use a value of 8 mg/L for C-reactive protein when calculating model probabilities of death (median value in the development cohort of the models). For other variables, data completeness was substantially better in the smaller UKRR-1 cohort (Table 1).

**Table 1:** Percentage of missing original data (prior to multiple imputation) with respect to the prediction models’ variables, and according to data set.

Variable	Finnish validation set (*n* = 1768)	NECOSAD^a^ (*n* = 2051)	UKRR-1 (*n* = 5328)	UKRR-2 (*n* = 45493)
Age at KRT start	0.0	0.3	0.0	0.0
ESKD diagnosis	0.0	0.0	1.5	3.3
Serum albumin	4.9	11.6	5.6	16.4
Serum phosphate	2.9	6.5	6.4	14.6
Serum C-reactive protein	9.1	58.8	100.0^b^	100.0^b^
Heart failure	5.5	11.0	6.8	56.0
Peripheral vascular disease	4.8	11.0	7.0	39.1
Peripheral vascular disease with limb amputation	3.6	11.1	6.9	38.6

^a^Percentage missing within the whole original cohort.

^b^Please see text.

Variables of the one-year model: Age at KRT start, primary kidney disease diagnosis, serum albumin, serum C-reactive protein, heart failure, serum phosphate, peripheral vascular disease.

Variables of the two-year model: Age at KRT start, primary kidney disease diagnosis, serum albumin, serum C-reactive protein, heart failure, peripheral vascular disease with limb amputation.

### Imputation for missing data

In order to include the entire Dutch and UK validation cohorts, we performed multiple imputation for missing data using the fully conditional specification [28]. We have provided a complete list of predictors used for imputation (S1 Appendix). We calculated the average probability of death individually for each patient as a mean of the result in all ten imputation sets.

### Statistical methods

The earlier published logistic regression models were used to calculate probabilities of death at one and at two years from start of dialysis for the patients in the NECOSAD and UKRR validation cohorts. The algorithms are shown in the (S2 Appendix). Discrimination of the models was evaluated using C-statistic (area under the receiver operating characteristic curve, AUC). Calibration was assessed using calibration plots (average estimated probability of death was plotted against observed risk of death in tenths of the probabilities). Two-sided P-values lower than 0.05 were considered statistically significant. For statistical analyses, we used PASW Statistics 25 (IBM SPSS Statistics for Windows. Armonk, NY: IBM Corp.).

## Results

### Study populations

There were some significant differences in baseline characteristics between the four validation groups (Table 2). Patients in the UKRR-1 and UKRR-2 cohorts were older than the Dutch or the Finnish patients, and the proportion of men was higher in the Finnish cohort. There were substantially more patients starting with peritoneal dialysis in the NECOSAD cohort, whereas hemodialysis was more frequent in both UKRR cohorts. Of primary kidney diseases, polycystic disease was less common in the UK patients and both type 1 and type 2 diabetes were more frequent among the Finns and pyelonephritis among the Dutch. In the UKRR-2 cohort, patients more often had heart failure.

**Table 2:** Baseline patient characteristics according to data set.

Characteristic	Finnish validation cohort (*n* = 1768)	NECOSAD (*n* = 2051)	UKRR-1 (*n* = 5328)	UKRR-2 (*n* = 45493)
Age at KRT start, yrs median (IQR)	64.0 (19)	63.0 (22.2)	68.2 (21.7)	65.9 (22.7)
Males, *n* (%)	1198 (67.8)	1271 (62.0)	3369 (63.2)	28622 (62.9)
Body mass index, kg/m^2^ median (IQR)	26.6 (7.3)	24.4 (5.1)	27.4 (8.1)	27.4 (8.1)
Initial dialysis modality, *n* (%)				
Hemodialysis	1336 (75.6)	1311 (63.9)	4316 (81.0)	36009 (79.2)
Peritoneal dialysis	432 (24.4)	734 (35.8)	1012 (19.0)	9484 (20.8)
Laboratory measurements, median (IQR)				
eGFR (CKD-EPI) at KRT start, ml/min/1.73m^2^	7.4 (4.0)	5.1 (4.0)	7.5 (3.7)	7.3 (3.8)
Blood hemoglobin, g/L	106 (20)	111 (21)	101 (19)	100 (20)
Serum albumin, g/L	32.0 (9.8)	36.0 (8.0)	34.0 (8.0)	34.0 (8.0)
C-reactive protein, mg/L	6 (17)	5 (11)	NA	NA
Serum phosphorus, mmol/L	1.82 (0.73)	1.76 (0.71)	1.49 (0.60)	1.46 (0.59)
Primary kidney disease diagnosis, *n* (%)				
Glomerulonephritis	241 (13.6)	252 (12.3)	732 (13.9)	5611 (12.8)
Polycystic disease	178 (10.1)	195 (9.5)	320 (6.1)	2613 (5.9)
Diabetes type 1	239 (13.5)	^a^	596 (11.4)	4284 (9.7)
Diabetes type 2	378 (21.4)	295 (14.4)	637 (12.1)	7224 (16.4)
Pyelonephritis	41 (2.3)	204 (9.9)	396 (7.5)	2762 (6.3)
Amyloidosis	47 (2.7)	20 (1.0)	85 (1.6)	572 (1.3)
Nephrosclerosis	113 (6.4)	181 (8.8)	405 (7.7)	3135 (7.1)
Other	255 (14.4)	361 (17.6)	1303 (24.8)	9860 (22.4)
Unknown	276 (15.6)	543 (26.5)	776 (14.8)	7919 (18.0)
Comorbidity^b^				
Angina pectoris	301 (18.0)	211 (11.6)	485 (10.1)	3055 (11.5)
Myocardial infarction	255 (15.0)	238 (13.0)	627 (13.1)	3472 (13.2)
Left ventricular hypertrophy	581 (36.8)	272 (14.9)	NA	NA
Heart failure	178 (10.7)	228 (12.5)	61 (12.4)	3028 (15.1)
Peripheral vascular disease	216 (12.8)	277 (15.2)	598 (12.1)	3463 (12.5)
Peripheral vascular disease with limb amputation	82 (4.8)	33 (1.8)	134 (2.7)	786 (2.8)
Stroke	198 (11.7)	156 (8.5)	480 (10.0)	2940 (11.1)
Cancer	217 (12.3)	177 (9.7)	757 (15.8)	3698 (13.9)

^a^Included in Diabetes type 2.

^b^Of the ones with data available.

KRT, kidney replacement therapy; IQR, interquartile range; eGFR, estimated glomerular filtration rate; CKD-EPI, Chronic Kidney Disease Epidemiology Collaboration.

Only a few patients experienced recovery of kidney function after three months from KRT start in any of the validation groups (Table 3). Number of kidney transplantations, however, differed greatly between the validation groups with fewer kidney transplantations performed in the NECOSAD patient cohort.

**Table 3:** Outcome according to data set.

	Finnish validation set (*n* = 1768)		NECOSAD (*n* = 2051)		UKRR-1 (*n* = 5328)		UKRR-2 (*n* = 45493)	
	During 1 year	During 2 years	During 1 year (*n* = 1964)	During 2 years (*n* = 1913)	During 1 year	During 2 years	During 1 year	During 2 years
Recovery of kidney function after 3 mo from KRT start, *n* (%)	24 (1.4)	25 (1.4)	15 (0.8)	25 (1.3)	53 (1.0)	14 (0.3)	399 (0.9)	123 (0.3)
Recipients of kidney transplant, alive at 1 and at 2 years, *n* (%)	104 (5.9)	226 (12.8)	54 (2.7)	98 (5.1)	336 (7.4)^a^	305 (7.7)^a^	2932 (7.6)^a^	2729 (8.1)^a^
Loss to follow-up, *n*	0	0	3	7	2	2	22	16
Mortality, all patients, *n* (%)	197 (11.1)	283 (21.2)	196 (10.0)	353 (18.5)	810 (15.2)	1381 (25.9)	7028 (15.4)	11686 (25.7)

KRT, kidney replacement therapy.

^a^Alive at the end of year 1 and at the end of year 2.

### Outcome: Mortality

Mortality rates were different between the validation groups, with lower one- and two-year mortality in the NECOSAD cohort and higher mortality in both UKRR cohorts (Table 3).

### Validation

Validation of the models in the NECOSAD cohort yielded even higher AUC values compared to the Finnish validation cohort: 0.79 in the one-year model and 0.78 in the two-year model (Table 4) (versus the Finnish cohorts’ AUCs: 0.77 and 0.74). The results for both UKRR data sets were somewhat lower: AUC 0.73 and 0.73 for one- and two-year mortality in the UKRR-1 cohort, and 0.74 and 0.74 in the larger UKRR-2 cohort.

**Table 4:** Predictive ability of the study models using c-statistic (area under the curve, AUC). Results of external validation according to data set.

	Finnish validation set (*n* = 1768)	NECOSAD (*n* = 2051)	UKRR-1 (*n* = 5328)^b^	UKRR-2 (*n* = 45493)^b^
One-year model	0.77 (*n* = 1418)^a^	0.79 (*n* = 1964)^b^	0.73	0.74
Two-year model	0.74 (*n* = 1101)^a^	0.78 (*n* = 1913)^b^	0.73	0.74

^a^The number of patients for whom data on all model variables were available.

^b^The number of patients after multiple imputation was performed.

We further validated the models separately for those patients who started dialysis treatment with HD and those with PD. Overall, the performance of the models was better in PD patients, AUCs ranging from 0.76 to 0.84 (Table 5).

**Table 5:** Predictive ability of the study models using c-statistic (area under the curve, AUC), stratified by the initial dialysis modality (HD or PD). Results of external validation according to data set.

	Finnish validation set, HD	Finnish validation set, PD	NECOSAD HD (*n* = 1311)^a^	NECOSAD PD (*n* = 734)^a^	UKRR-1 HD (*n* = 4316)^b^	UKRR-1 PD (*n* = 1012)^b^	UKRR-2 HD (*n* = 36009)^b^	UKRR-2 PD (*n* = 9484)^b^
One-year model	0.75 (*n* = 1093)	0.78 (*n* = 360)	0.75	0.84	0.71	0.81	0.72	0.78
Two-year model	0.72 (*n* = 819)	0.78 (*n* = 281)	0.74	0.82	0.71	0.76	0.72	0.78

^a^There were 6 patients with missing information on initial dialysis modality in the NECOSAD cohort.

^b^Data on initial dialysis modality were available for all the patients of the UKRR-1 and UKRR-2 cohort.

**Fig 1:**
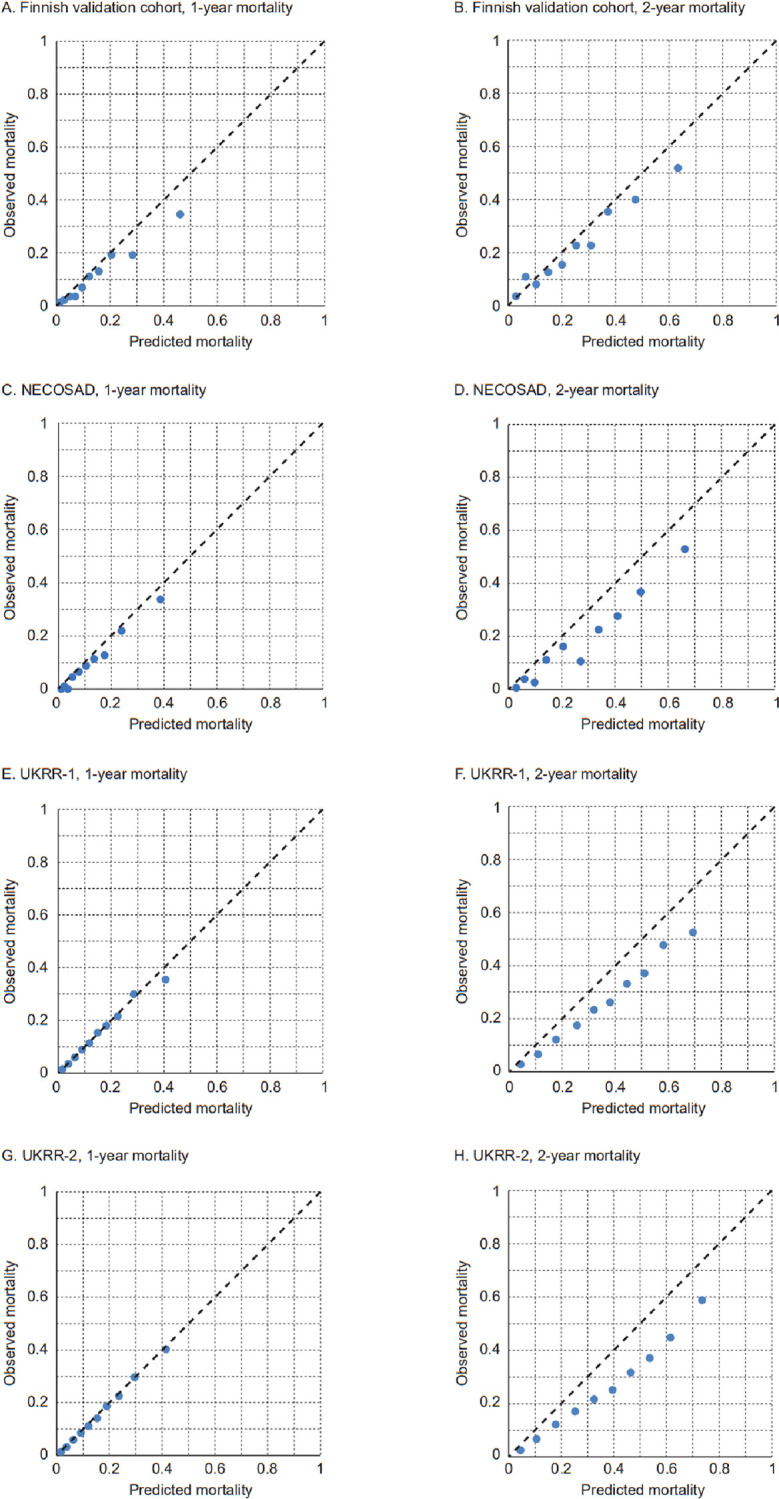
Calibration plots for one- and two-year mortality per decile of predicted mortality in the original Finnish validation cohort (A-B) and comparison to the NECOSAD cohort (C-D) and to both UKRR cohorts (E-H).

To assess calibration, the calculated probabilities of death at one and at two years after KRT start for each patient were ranked and divided into tenths. Average probabilities in the tenths were then plotted against observed risk of death (Fig 1). The graphical presentation indicated good calibration for the one-year mortality prediction in all validation cohorts, whereas the two-year model somewhat overpredicted the risk in all cohorts (i.e., death rates were, in reality, lower than our models predicted).

## Discussion

In the present study, we performed external validation of two earlier published Finnish mortality prediction models in patients who started dialysis in the Netherlands and the United Kingdom. Overall, these models, built to predict one- and two-year mortality after start of long-term dialysis, showed good discrimination with AUC of 0.73 to 0.79. The calibration of the one-year mortality model was good, whereas mortality was somewhat overestimated by the two-year model.

Several prognostic models for patients starting dialysis already exist. These models are usually rather similar with regard to type of predictive variables that are included, but the number of variables vary. Most models, also ours, include patient’s age, serum albumin and primary kidney disease, all of which are known to have strong influence on KRT patients’ prognosis, and additionally some comorbidities are often included [12,16]. The number of variables affects the usability of a model. Some earlier models were based on extensive lists of variables, but during recent years researchers have attempted to limit the number of predictors [18,29]. On the other hand, this is likely to weaken a model’s performance, and thus deciding on the number of predictors means balancing between model practicality and predictive performance. Our one- and two-year models include seven and six variables, respectively, a low number compared to most published models [12,16,20], and are therefore relatively easy to use.

Even more important than practicality is the predictive performance of the model. In most studies, model validation has been done by assessing discrimination with AUC, and according to a recent meta-analysis most models reached on average a value of 0.71, a result which may be regarded as reasonable [12]. Some models possess even better performance with an AUC over 0.80, suggesting good discrimination, but to achieve this a large number of predictors are usually needed [12]. Validation of a model by evaluating calibration is another central process and reflects number of events predicted by a model against number of observed real-life events [15]. Unfortunately, calibration has not been reported for many of the published models [12]. For those reported, most recent models have tended to overestimate risk of death especially when applied to more recent patient populations than the one used for model construction. This is likely the result of improved overall patient prognosis along the years.

When evaluating performance of any prediction model it is crucial to validate it externally [13,15,30]. By doing this one can objectively investigate a models’ broader validity and thus feasibility to clinical work. Predictive performance may be tested on a national level in a new patient population (for example, temporal or geographical), or in an even more demanding environment, in a patient population of another country. This has been done for only a few models that predict mortality of KRT patients. Hemke and colleagues validated a Dutch model on data from the ERA-EDTA Registry, including dialysis starters (*n* = 136304) between 1995 to 2005 from 10 different European countries [18]. This four-variable model (age, gender, primary kidney disease, and mode of dialysis 90 days from KRT start), designed to predict ten-year mortality, performed adequately in internal validation (AUC 0.72) [9]. In the external validation, they evaluated 10- (primary endpoint), five- and three-year survival prediction: the model had an AUC of 0.71 in the complete ten-country cohort, with varying performance between countries (AUCs 0.70–0.75). The model slightly overestimated the death risk in the external validation sets [18]. Ramspek and colleagues externally validated seven prediction models from six countries in their systematic review [16]. Notably, our models were not included in this review as they were published later, and thus we see it important to have done the validation now in this study. In the Ramspek et al. review, the performance of the seven models was tested in the NECOSAD study population (*n* = 1943), reaching AUC from 0.71 to 0.75 for predicting one-year mortality. Overall, the performance of the models was weaker in the external validation than what it had been in the original population. Furthermore, the performance varied not only between countries, but also with respect to study period, the poorest performance seen expectedly in the population temporally most distant from the validation cohort. Forzley and colleagues evaluated performance of a six-month mortality prediction model constructed with a US ESKD patient cohort (*n* = 512) externally in a Canadian ESKD cohort during 2006 to 2007 [19]. The model had a good degree of discrimination (AUC 0.80) in their original, partly external validation (*n* = 514). The performance of the model was, however, substantially weaker in another temporal (earlier) Canadian cohort (*n* = 374), with AUC of 0.70.

Predictive performance of our models in terms of discrimination may be regarded as good. Rather unexpectedly, AUC in the Dutch cohort was even better than in the Finnish validation group (AUC 0.79–0.78 versus 0.77–0.74) (Table 4). This might be due to the fact that the Dutch cohort comprised patients from an era closer to the era of the Finnish development cohort as compared to the Finnish validation cohort. Furthermore, some characteristics of the Dutch cohort resembled more the development group than the Finnish validation group (Table 2, results partly shown). On the other hand, there were also notable differences. For instance, the time-point of most laboratory data collection was about three months later in the Dutch cohort. Consequently, improvements in the laboratory test results caused by three months on dialysis would have been expected to reduce our models’ performance. Regarding calibration, our models overestimated risk of death at two years both in the NECOSAD and the UKRR cohorts, but interestingly, rather similarly despite the fact that mortality was significantly higher in the UKRR cohorts. This was probably a consequence of the higher age of the patients in the UKRR cohorts, which our models took into account. Of note, under- or overestimation of this sort could be solved by simple recalibration of the baseline risk [15,31].

Interestingly, with regard to discrimination, our models performed better in PD patients compared to HD patients. Furthermore, AUCs in the NECOSAD and UKRR PD populations were equal or even higher than in the Finnish validation set, with the only exception of two-year prediction in the UKRR-1 cohort. There may be several potential explanations to this, for instance, temporal resemblance of cohorts as well as similarities in patient characteristics between cohorts or even wider distribution of patient characteristics within a cohort. PD patients are in general younger with fewer comorbidities compared to HD patients, and this smaller interpatient variability may potentially have caused some of the differences seen in predictive performance of our models. However, based on our analyses we cannot fully explain the differences of validation results between PD and HD patients. The models performed better in PD patients already in the original Finnish validation set, and this better performance was then seen through the Dutch and UKRR validation cohorts. Acknowledging this, maybe constructing prediction models separately for PD and HD patients in the future could improve our ability to predict outcome of our dialysis patients.

There are some limitations as well as strengths of our study that we want to point out. First, the NECOSAD and the UKRR data sets were not complete with regard to all the variables of the Finnish models. However, we performed multiple imputation for missing data and thus prevented a substantial proportion of the patients from being excluded. This might have distorted our study results, but assuming that the values were missing at random, the results should be unbiased. In general, imputation is preferable to omitting patients with missing values from the validation [28]. Second, non-available data on serum C-reactive protein in the UK cohorts could not be imputed (not missing at random). Availability of complete data might have improved the models’ performance among the UK patients, and lack of these data may to an extent explain the slightly worse performance of our models in the UKRR population, compared to the NECOSAD population. Third, diabetes types 1 and 2 were combined in the NECOSAD data set and could not therefore be analyzed separately. However, because the proportion of patients with diabetes 1 type is very low in the Netherlands, we chose to consider all NECOSAD patients with diabetes to have type 2 diabetes. Fourth, the NECOSAD cohort was to a point selected (patients took part in the study voluntarily), compared to the UKRR cohorts which included all incident new dialysis starters. Fifth, although our models may be applied on patients entering long-term KRT in any country, the models have so far been validated only in countries with rather similar economies and health-care systems, and thus the robustness of the models in less similar countries is not known.

The obvious strength of this study is the international validation within three different ESKD cohorts from three countries, showing good performance of our models. This encourages to put these models to a real test by implementing them to clinical work and treatment decisions not only in Finland, but in other European countries as well. Preferably, the models should be tested in an impact study.

In all earlier published studies, and ours as well, performance of different mortality prediction models has been compared only “on paper”: by their capabilities in discrimination and calibration, and not by how their use could possibly affect patient outcomes. The next step would be to explore how these models could assist in clinical management of ESKD patients, and ultimately, in improving their prognosis. Which could then be the ways to implement models to everyday clinical work? Certainly, usability plays an important role and favors models with fewer variables. A model connected to electronic patient medical chart could automatically provide the clinician with the model’s prediction. Furthermore, an easy-access internet-based model might encourage wider international testing and use of the models. For that purpose, we have recently constructed an open-access webpage, on which our prediction models can be easily used and implemented in management of ESKD patients (https://dev.arrak.fi/finne/rrt.html).

## Conclusions

A reliable mortality prediction model may provide important information for critical resource-related and patient-level decisions. Kidney patients and nephrologists need information about prognosis to make informed decisions about treatments, so that the treatments will be justified, sound, and the ones patients wish to receive. Our one- and two-year mortality prediction models, which have now been validated internationally, have shown good performance across differing patient populations, are easy to use and ready to be tested internationally in practice. Our plan is next to critically evaluate whether clinical use of these models improves treatment and outcomes of our patients.

## Supporting information

S1 Appendix Predictor variables used in imputation for missing data. (DOCX)

S2 Appendix Algorithms for mortality prediction. Calculation of mortality risk at one or two years from start of kidney replacement therapy.
(DOCX)

S1 File (DOCX)
