# Supplementary material for: Predicting mortality after start of long-term dialysis–International validation of one- and two-year prediction models
Source: PLoS One. 2023 Feb 22;18(2):e0280831. doi: 10.1371/journal.pone.0280831 (PMC9946236; doi:10.1371/journal.pone.0280831)
Supplement: S1 Appendix — (DOCX) [file pone.0280831.s001.docx]

S1 APPENDIX. Predictor variables used in imputation for missing data.

NECOSAD data set:

Gender, age at kidney replacement therapy (KRT) start, primary kidney disease, start year of KRT, blood haemoglobin, serum albumin, serum phosphorus, serum C-reactive protein, body mass index, systolic blood pressure, diastolic blood pressure, heart failure, peripheral vascular disease, peripheral vascular disease with limb amputation, Davies score, Charlson index, dead or alive at one year from KRT start, dead or alive at two years from KRT start.

UK Renal Registry data set:

Gender, age at KRT start, start year or KRT, dialysis modality at KRT start, primary kidney disease, blood haemoglobin, serum albumin, serum phosphorus, height, weight, systolic blood pressure, diastolic blood pressure, peripheral vascular disease, peripheral vascular disease with limb amputation, angina pectoris, previous coronary artery bypass graft or angioplasty, previous non-coronary stenting, angioplasty or vascular graft, previous myocardial infarction within last three months, previous myocardial infarction over three months ago, claudication, chronic obstructive pulmonary disease, diabetes, ischaemic or neuropathic ulcer, liver disease, malignancy, cerebrovascular disease, current or previous smoker, dead or alive at one year from KRT start, dead or alive at two years from KRT start.
