# Supplementary material for: Predicting mortality after start of long-term dialysis–International validation of one- and two-year prediction models
Source: PLoS One. 2023 Feb 22;18(2):e0280831. doi: 10.1371/journal.pone.0280831 (PMC9946236; doi:10.1371/journal.pone.0280831)
Supplement: S2 Appendix — Calculation of mortality risk at one or two years from start of kidney replacement therapy. (DOCX) [file pone.0280831.s002.docx]

S2 APPENDIX. Algorithms for mortality prediction.

Risk = 1/(1 + exp(–L))

For calculating one-year mortality:

L = –4.624 +0.0488**Age* +*ESKD Diagnosis* –0.0419**Serum albumin* (g/l) + *Serum phosphate* +0.1484*ln(*C-reactive protein*, mg/l) +0.7419 (if *heart failure*, otherwise 0) +0.5068 (if *peripheral vascular disease*, otherwise 0)

*ESKD Diagnosis* = 0 for glomerulonephritis, –0.5798 for polycystic kidney disease, 0.7701 for type 1 diabetes, 0.4886 for type 2 diabetes, 0.1310 for pyelonephritis, 1.1314 for amyloidosis, 0.3920 for nephrosclerosis, 0.8671 for other specified diagnoses, and 0.3988 for unknown diagnosis

*Serum phosphate* = 0 if <1.53 mmol/l, –0.2877 if 1.53–<2.0 mmol/l, and 0.1398 if >2.0 mmol/l

For calculating two-year mortality:

L = –4.073 +0.0583**Age* +*ESKD Diagnosis* –0.0408**Serum albumin* (g/l) +0.1044*ln(*C-reactive protein*, mg/l) +0.9083 (if *heart failure*, otherwise 0) +0.6419 (if *peripheral vascular disease with limb amputation*, otherwise 0)

*ESKD Diagnosis* = 0 for glomerulonephritis, –0.3147 for polycystic kidney disease, 1.033 for type 1 diabetes, 0.7747 for type 2 diabetes, –0.2357 for pyelonephritis, 1.3137 for amyloidosis, 0.4824 for nephrosclerosis, 0.8416 for other specified diagnoses, and 0.4121 for unknown diagnosis
